# Fat mass and obesity‐associated protein regulates tumorigenesis of arecoline‐promoted human oral carcinoma

**DOI:** 10.1002/cam4.4188

**Published:** 2021-08-11

**Authors:** Xia Li, Xiaoli Xie, Yangcong Gu, Jianming Zhang, Jiang Song, Xiufeng Cheng, Yijun Gao, Yilong Ai

**Affiliations:** ^1^ Department of Oral Medicine Foshan Stomatological Hospital Medical College of Foshan University Foshan China; ^2^ Department of Endodontics Hunan Xiangya Stomatological Hospital Central South University Changsha China; ^3^ Department of Oral Maxillofacial Surgery Foshan Stomatological Hospital Medical College of Foshan University Foshan China; ^4^ Department of Preventive Dentistry Foshan Stomatological Hospital Medical College of Foshan University Foshan China; ^5^ Department of Stomatology The Second Xiangya Hospital Central South University Changsha China

**Keywords:** arecoline, FOXA2, FTO, oral carcinoma, tumorigenesis

## Abstract

Arecoline, a major alkaloid within areca nut extract, is recognized as the primary active carcinogen promoting oral squamous cell carcinoma (OSCC) pathological development. Dysregulation of N6‐methyladenosine (m6A) methyltransferase components (e.g., Fat mass and obesity‐associated protein [FTO] and methyltransferase‐like 3 [METTL3]) are closely associated with multiple cancer progression, including oral cancer. However, the biological function role of FTO in arecoline‐induced oral cancer is largely unknown. We identified that FTO was significantly upregulated in OSCC tissues from patients with areca nut chewing habits and chronic arecoline‐treated OSCC cell lines. Depletion of FTO attenuated the arecoline‐promoted stemness, chemoresistance, and oncogenicity of OSCC cells. Finally, we revealed that FTO was negatively regulated by a transcription factor forkhead box protein A2 (FOXA2) in OSCC cells. This study, for the first time, demonstrated that FTO plays an oncogenic role in arecoline‐induced OSCC progression. Thus, developing new therapeutic agents targeting FTO may serve as a promising method to treatment OSCC patients, especially those with areca nut chewing habits.

## Graphical abstract

We identified that FTO was significantly upregulated in OSCC tissues from patients with areca nut chewing habits and chronic arecoline‐treated OSCC cell lines. Depletion of FTO attenuated the arecoline‐promoted stemness, chemoresistance, and oncogenicity of OSCC cells. Therapeutic agents targeting FTO may serve as a promising method to treat OSCC patients, especially those with areca nut chewing habits.

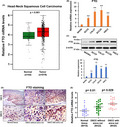

## 1 INTRODUCTION

Oral cancer ranks as the sixth most common cancer worldwide.^1^ Oral cancer is highly prevalent in Southeast Asia, such as India, Malaysia, Indonesia, and China.^2^ According to the estimation from the world health organization, there are over 657,000 new cases of oral cavity and pharynx cancers each year, and more than 330,000 deaths worldwide (https://www.who.int/cancer/prevention/diagnosis‐screening/oral‐cancer/en/). Oral cancer is particularly dangerous because it may not be noticeable in the early stages, as it frequently presents without pain or symptoms, and most oral cancer cases are diagnosed in the late stages.^3^, ^4^ In the late stages, oral cancer often has metastasized to other organs, such as cervical lymph nodes, liver, lung. It is highly lethal with a 5‐year survival rate of around 50%.^5^ Thus, to identify critical gene involved in tumorigenesis of oral cancer is extremely important for developing new targeting strategy for oral cancer treatment.

Carcinogens, including tobacco, alcohol, and areca nut, play a critical role in the initiation, development, and progression of oral cancer.^6^ Accumulating studies reveal a close association of areca nut exposure with oral cancer. Approximately 85% of oral cancer patients have areca nut chewing habit in Taiwan.^7^ The areca nut chewers exhibit a significantly higher risk of acquiring oral cancer than the non‐chewers. The 5‐year survival rate is much lower in oral cancer patients with areca nut chewing habit.^8^ Arecoline is the most abundant alkaloid within areca nut extract and is recognized as the main active carcinogen related to oral cancer pathological development.^9^ Arecoline is known to have mutagenic and genotoxic properties, which may contribute to the development of oral cancer.^7^, ^10^ However, the various effects of arecoline on oral cell transformation and the underlying molecular mechanisms are not fully understood.

Dysregulation of oncogenes and tumor‐suppressing genes is the hallmarks of cancer. DNA modifications have been well‐characterized as an impotent epigenetic regulation mechanism, contributing to cancer formation and development.^11^ In recent years, RNA N6‐methyladenosine (m6A) modifications are emerging frontiers in cancer biology research. M6A modification is generally mediated by m6A methyltransferase components and is reported to play a pivotal role in the regulation of various cellular functions through influencing target mRNA splicing, stability, and translation efficiency.^12^ M6A methyltransferase components mainly compose three types of enzymes, which are “writers” (e.g., methyltransferase‐like 3 [METTL3], METTL14, and Wilms tumor 1 [WT1]‐associated protein [WTAP]), “erasers” (e.g., obesity‐associated protein (FTO), alkB homologue 5 (ALKBH5), and ALKBH1), and “readers” (e.g., YT521‐B homology [YTH] domain family proteins).^13^ Recent studies demonstrated that some of these genes are involved in oral cancer development.^14^ However, the role of m6A modification gene in arecoline‐mediated oral cancer transformation and development has never been investigated.

In the present study, we identified that FTO was significantly upregulated in oral squamous cell carcinoma (OSCC) tumor samples from patients with areca nut chewing habit and in chronic arecoline‐treated oral cancer cell lines, highlighting the potentially critical role of FTO in oral cancer progression. Elucidating the biological function role of FTO in arecoline‐promoted oral cancer progression is urgently needed.

## 2 MATERIALS AND METHODS

### 2.1 Patient samples

A total of 42 OSCC tissues and 18 normal tissues (oral mucosa tissues) were collected in the Foshan Stomatology Hospital and Chenzhou No. 1 People's Hospital during 2015 to 2017. Among 42 OSCC tissues, 20 OSCC tissues were obtained from patients with areca nut chewing habit and the rest were from the patients who never chewed areca nut. All tissues were stained with H&E and were independently examined by two pathologists.

### 2.2 Ethics statement

All patients provided written informed consent and the experimental procedures were approved by the Institutional Review Board of the Ethics Committee of the Foshan Stomatology Hospital.

### 2.3 Cell culture

SCC25, SCC9, and CAL27 were purchased from American Type Culture Collection (ATCC, VA, USA). Immortalized normal human oral keratinocytes (NHOK), 293T, and HSC2 cell line were purchased from (Cell Bank of Type Culture Collection of Chinese Academy of Sciences, Shanghai, China). 293T cell line and OSCC cell lines (SCC25, SCC9, HSC2, and CAL27) were cultured in Dulbecco's modified Eagle's medium (DMEM) supplemented with 2.5 mM L‐glutamine, 15 mM HEPES, 0.5 mM sodium pyruvate, and 10% fetal bovine serum (FBS). NHOK cell line was cultured in EpiLife (M‐EPI‐500‐CA) with the addition of human keratinocyte growth supplement (ThermoFisher Scientific, CA, USA). Cells were maintained in an incubator at 37℃ with an atmosphere at 5% CO_2_. To establish the long‐term arecoline‐treated OSCC cell lines, SCC25 and CAL27 were cultured in the complete DMEM medium supplemented with DMSO as a control, or arecoline (1 μg/ml or 2.5 μg/ml) (Millipore Sigma, St. Louis, MO, USA). The medium was replaced every 3 days and cells were passaged every 6 days of culture. The arecoline treatment lasted for 90 days and cells were passaged 15 times.

### 2.4 Quantitative reverse transcription PCR (RT‐qPCR)

Tissues were snap‐frozen in liquid nitrogen and were ground into small pieces (1 mm^3^) and lysed in TRIzol reagent (Invitrogen). OSCC cells were washed and lysed in TRIzol reagent. Total cDNAs were synthesized using a SuperScript III First‐Strand Synthesis System. The cDNAs were mixed with primers and Universal SYBR® Green Supermix (BioRad, Hercules, CA, USA), and run PCR reaction on ABI Prism 7500 System (Applied Biosystems, CA, USA). The relative gene expression data were normalized to the GAPDH. The primers are listed in the Table S1.

### 2.5 Immunohistochemistry

OSCC tumor tissues or normal oral tissues were fixed in Bouin's solution, washed, and embedded in paraffin. Tissue sections underwent deparaffinization procedure in a series of xylene and ethanol. The heat‐mediated antigen retrieval was performed using sodium citrate buffer (10 mM Sodium citrate, 0.05% Tween 20, pH 6.0). The sections were treated with 0.3% H_2_O_2_, and then blocked in blocking buffer for 2 h. The sections were incubated with primary antibody (anti‐FTO antibody [EPR6894] (ab126605) for human tissues, and anti‐ FTO antibody [EPR6895] (ab124892) for mouse tissues) overnight in a cold room, and then applied with horseradish peroxidase (HRP)‐conjugated secondary antibody. The sections were immersed in (3,3′‐diaminobenzidine) DAB reagent, mounted, and coverslipped.

### 2.6 Western blotting assay

OSCC cells were lysed in lysis buffer containing protease and phosphatase inhibitors. Protein samples of 20–30 µg were loaded into sodium dodecyl sulphate–polyacrylamide gel and separated by electrophoresis. After the proteins were transferred onto polyvinylidene difluoride (PVDF) membrane, the membrane was probed with primary antibodies, followed by second antibodies. The protein signals were developed after incubation with chemiluminescent substrates. Antibodies against KLF4 (D1F2) (12173S, 1:1000 dilution), Nanog (D2A3) (8822S, 1:1000 dilution), Sox2 (D6D9) (3579S, 1:1000 dilution), and β‐actin (3700S, 1:5000 dilution) were purchased from Cell Signaling Technology (MA, USA). Anti‐FOXA2 [EPR4466] (ab108422, 1:1000 dilution), and anti‐FTO antibody [EPR6894] (ab126605, 1:1000 dilution) were from Abcam (Cambridge, UK).

### 2.7 Cell proliferation assay

Cell viability was measured by using cell counting kit‐8 (CCK8) assay according to manufactory's instruction. Briefly, cells were grown in 96‐well plates and 10 µl of WST‐8 solution was added. The plate was incubated in an incubator at 37℃ for 4 h. The absorbance of each well was measured using a microplate reader at 460 nm.

For colony formation assay, OSCC cells (3000 cells/well) were mixed with 0.5% soft agar and seeded into 6‐well plate. Cells were maintained in an incubator for 14‐day. After that, cells were fixed with 100% methanol and staining with 0.5% crystal violet. Colony number was counted under an inverted microscope.

### 2.8 Cell migration assay

OSCC cells were seeded in the up‐layer of Boyden Chamer insert (8 μm pore‐size) with serum‐free medium. The insert was placed into the well of 24‐well plate with complete medium in the well. After cultured in an incubator for 24 h, the up‐layer cells were removed and the migrated cells at the bottom of the insert were fixed. Then the cells were stained with 0.5% crystal violet and the migrated cells were count under a microscope.

### 2.9 Flow cytometry

OSCC cells were washed and resuspended in PBS buffer with 2% FBS. Cells were blocked with Fc‐block buffer for 15 mins and then incubated with anti‐ALDH [5A11] (ab105920) (1:100 dilution) for 30 mins in 4℃. Cells were washed with PBST twice and resuspend cells in 300 µl for flow cytometric analysis (Cytek™ Aurora).

### 2.10 Enzyme‐linked immunosorbent assay (ELISA)

The expression levels of TNF‐α (ELH‐TNFα‐1), IFN‐γ (ELH‐IFN‐γ‐1), IL‐2 (ELH‐ IL‐2–1), GM‐CSF (ELH‐ GM‐CSF‐1), IL‐10 (ELH‐IL‐10–1), IL‐4 (ELH‐IL‐4–1), TGF‐β (ELH‐TGF‐β‐1), and IL‐17 (ELH‐IL‐17–1) in tumor tissues were determined using the ELISA kit purchased from Raybiotech (Peachtree Corners, GA, USA). The tumor lysate samples were diluted for 10‐fold with sample diluent buffer to yield 1 mg of protein per 1 ml of original lysate solution. Then the samples (100 μl) were added into the 96‐well plated pre‐coated with appropriate antibody overnight. The plate was washed and incubated with biotinylated antibody, followed by Streptavidin solution. The signal was developed with 3,3′,5,5′‐tetramethylbenzidine (TMB) one‐step substrate reagent. The absorbance of each well was measured using a microplate reader at 450 nm.

### 2.11 Lentiviral particles and adenoviral particles

Lentiviral particles carrying FOXA2 gene (LPP‐Y4558‐Lv105‐050‐S), shFOXA2 (Short hairpin RNA targeting FOXA2) (LPP‐HSE061179‐LVE004‐050), shFTO (Short hairpin RNA targeting FTO) (LPP‐HSH111734‐LVRH1H‐050), and control lentivirus (LP129‐025) were purchased from GeneCopoeia (MD, USA).

Adenoviral particles carrying FTO cDNA (AD05‐H1661‐EB001‐A00), or control adenoviral particles (EN010‐100) were obtained from GeneCopoeia (MD, USA).

### 2.12 Luciferase reporter assay

A PCR product containing the 5′ untranslated region (UTR) region of the FTO gene (−200 bp to +34 bp) with Kpn I and Bgl II restriction enzymes recognition sites was amplified from 293T cells DNA. The PCR product was digested and ligated into pGL3‐basic vector (pGL3‐FTO) (Promerga, WI, USA). The plasmid was sequenced to confirm the sequence of the insert. For luciferase reporter assay, 293T cells were transduced with indicated lentiviral particles and then co‐transfected with pGL3‐FTO and pRT‐TK vectors (Promerga) using lipofectamine 2000 (ThermoFisher Scientific, CA, USA) according to manufacturer's instructions. After 24 h, the luciferase activities were determined using the Dual Luciferase Reporter Assay System Kit (Promega) in GloMax® 20/20 Luminometer (Promerga). The primers are listed as following: pGL3‐FTO F: 5′‐GCGGGTACCTCCACCCACCCTCATCCTCC‐3′; pGL3‐FTO R: 5′‐GCCAGATCTGAATTTCCCAGGTCCGT‐3′.

### 2.13 Animal model

Thirty BALB/c strain nude mice were purchased from Guangdong Medical Science Experiment Center (Guangdong, China). Mice were randomly divided into three groups (*n* = 10 for each group), and were maintained in specific‐pathogen‐free (SPF) animal facility. Mice were subcutaneously injected with CAL27‐shRNA, CAL27‐A‐shRNA, or CAL27‐A‐shFTO, and tumor volumes were measured every 3 days starting at 10‐day post tumor implantation. Tumor volumes were calculated with the formula: V = (length ×width × width) × 0.5. Mice were sacrificed on 31‐day post tumor implantation. Tumor tissues were collected and weighted. Animal experiments were performed in accordance with the guide for the care and use of laboratory animals. The animal experimental protocol was approved by the Ethics Committee of the Foshan Stomatology Hospital.

### 2.14 Statistical analysis

Data are presented as means ±SEM. Unpaired two‐tailed Student's *t*‐test or one‐way analysis of variance (AVONA) was applied to compare differences between two groups or more than two groups, respectively. Spearman rank‐order correlation analysis was used to test the relationship between FTO and FOXA2. Statistical analyses were performed using Graphpad Prism 8 (CA, USA). *p* values <0.05 were considered statistically significant.

## 3 RESULTS

### 3.1 Chronic arecoline treatment enhances FTO expression in OSCC cell lines

**FIGURE 1:**
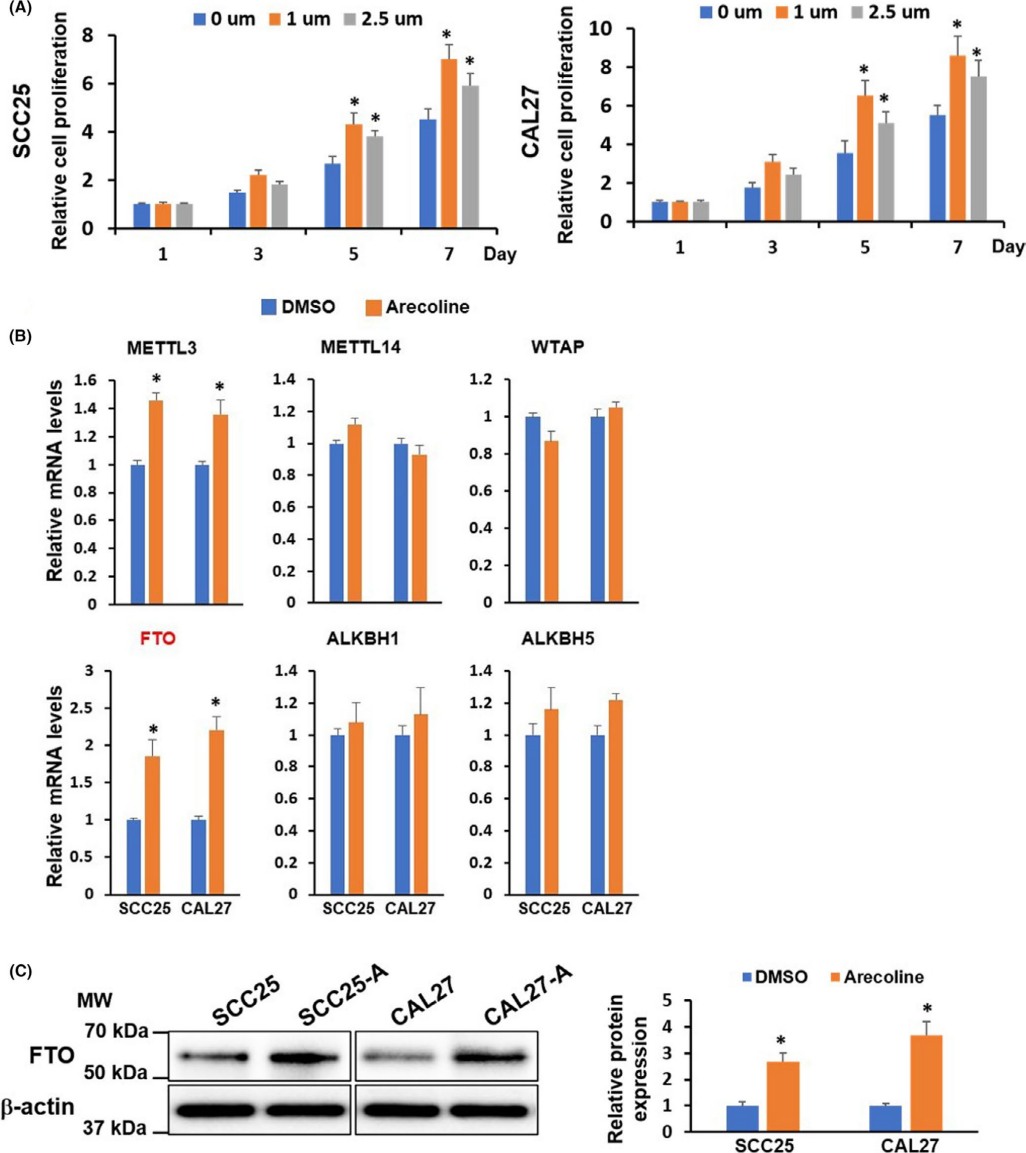
FTO is significantly upregulated in long‐term arecoline‐treated OSCC cell lines. (A) SCC25 and CAL27 cells were treated with DMSO as a control, or arecoline (1 μg/ml or 2.5 μg/ml) for 90 days to establish long‐term arecoline‐treated OSCC cell lines. The cell proliferation of these cells on 1‐, 3‐, 5‐, and 7‐day were determined by CCK‐8 assay. (B) The expression levels of METTL3, METTL14, WTAP, FTO, ALKBH1, and ALKBH5 in long‐term DMSO‐ or arecoline (1μg/ml)‐treated OSCC cell lines were detected by RT‐qPCR assay. (C) The protein levels of FTO in long‐term DMSO‐ or arecoline (1μg/ml)‐treated OSCC cell lines were measured by western blotting assay, and were quantified by Image J. Results are presented from three independent experiments. The molecular weight (MW) was depicted in the Figure. *p < 0.05 versus DMSO control group.

To investigate the chronic tumorigenic effect of arecoline on oral cancer cells, we initially treated the OSCC cell lines (SCC25 and CAL27) with various concentrations of arecoline for different time points and measured the cell viability and proliferation of OSCC cells. We found that high concentration (above 50 µg/ml) of arecoline caused acute cytotoxicity, resulting in OSCC cell growth arrest or cell death within 72 h (Figure S1A,B). Thus, a high concentration of arecoline is not suitable for long‐term arecoline treatment. Then, OSCC cell lines were exposed to increasing low concentrations of arecoline for 7‐day and 14‐day. We found that arecoline at 1 µm and 2.5 µm enhanced cell proliferation of SCC25 and CAL27 after 7‐day and 14‐day treatment (Figure S1C,D). Thus, these two doses were chosen to treat SCC25 and CAL27 cell lines for at least 3 months, approximately 15 cell‐passaging to establish chronic arecoline exposure OSCC cell lines. As depicted in Figure 1A, the chronic arecoline treatment at 1 µm yielded a stronger cell growth promotion effect than that at 2.5 µm. Thus, the 1 µm arecoline exposure OSCC cell lines were used for the following experiments (hereinafter referred to as SCC25‐A and CAL27‐A) (Figure 1A). To explore which m6A modification elements are involved in arecoline‐induced tumor‐promoting effects on OSCC, we compared the expression levels of several m6A modification key elements between arecoline‐treated and DMSO‐treated OSCC cell lines. The results showed that the expression levels of FTO and METTL3 were significantly upregulated in both SCC25‐A and CAL27‐A compared to SCC25 and CAL27, suggesting that m6A modification elements might play an important role in arecoline‐mediated OSCC progression (Figure 1B,C). FTO was selected for further study because FTO was the most upregulated m6A modification gene after long‐term arecoline treatment in OSCC cells.

### 3.2 FTO is upregulated in HNSCC and OSCC tissues and cell lines

**FIGURE 2:**
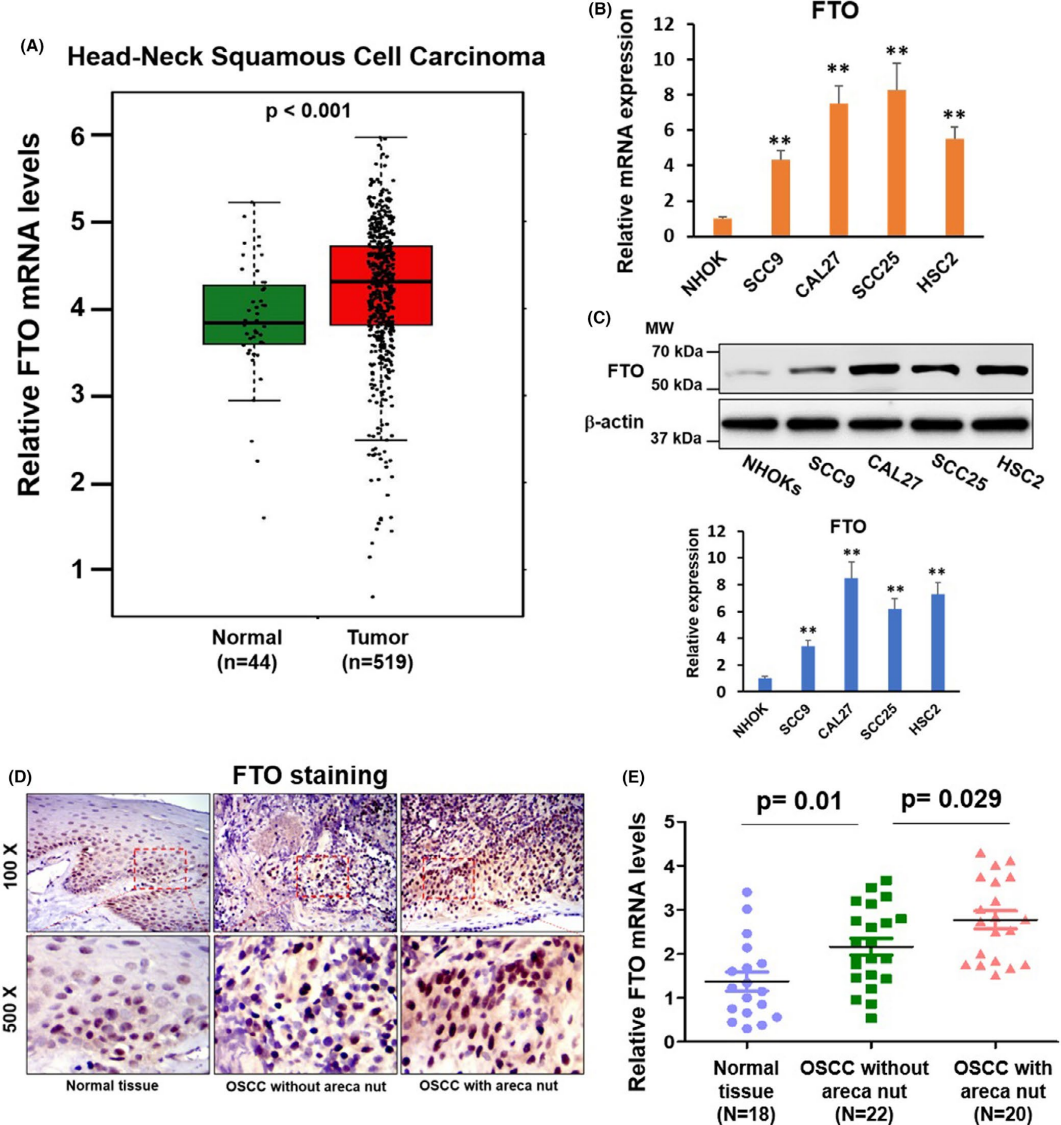
FTO is markedly elevated in OSCC tissues, especially from patients with areca nut chewing habit. (A) The expression levels of FTO in normal tissues (*n* = 44) and HNSCC tissues (*n* = 519). The results were collected and analyzed from TCGA (http://software.broadinstitute.org/software/igv/tcga) database. (B) The expression levels of FTO in NHOK, SCC9, CAL27, SCC25, and HSC2 cells were detected by RT‐qPCR assay. (C) The protein levels of FTO in NHOK, SCC9, CAL27, SCC25, and HSC2 cells were measured by western blotting assay, and were quantified by Image J. Results are presented from three independent experiments. **p < 0.01 versus NHOK. (D,E) The expression levels of FTO in normal oral tissues (*n* = 18), OSCC without areca nut (*n* = 22), and OSCC with areca nut (*n* = 20) were determined by IHC staining (D), and RT‐qPCR assay (E). The molecular weight (MW) was depicted in the Figure. *p < 0.05 versus control group

Through bioinformatics analysis using The Cancer Genome Atlas (TCGA; http://software.broadinstitute.org/software/igv/tcga) databases, we found that FTO mRNA levels were substantially upregulated in head and neck squamous cell carcinoma (HNSCC) tumor samples (*n* = 519) when compared with that in normal tissues (*n* = 44) (Figure 2A). The upregulation of FTO mRNA and protein levels was also observed in four OSCC cell lines compared to normal oral keratinocytes (NHOKs) (Figure 2B,C). Furthermore, we analyzed FTO expression in 18 normal tissues (oral mucosa tissues) from healthy donors without areca nut chewing habit and 42 OSCC tumor samples collected from OSCC patients, in which 20 had areca nut chewing habit. As illustrated in Figure 2D,E, the FTO mRNA and protein levels were higher in OSCC tumor samples than in healthy tissues. More importantly, the FTO levels were markedly upregulated in OSCC samples with areca nut exposure compared to OSCC samples without areca nut exposure. These results collectively suggested that FTO may involve not only in OSCC tumorigenesis, but also in arecoline‐mediated OSCC progression.

### 3.3 FTO plays a crucial role in arecoline‐promoted OSCC cellular function

**FIGURE 3:**
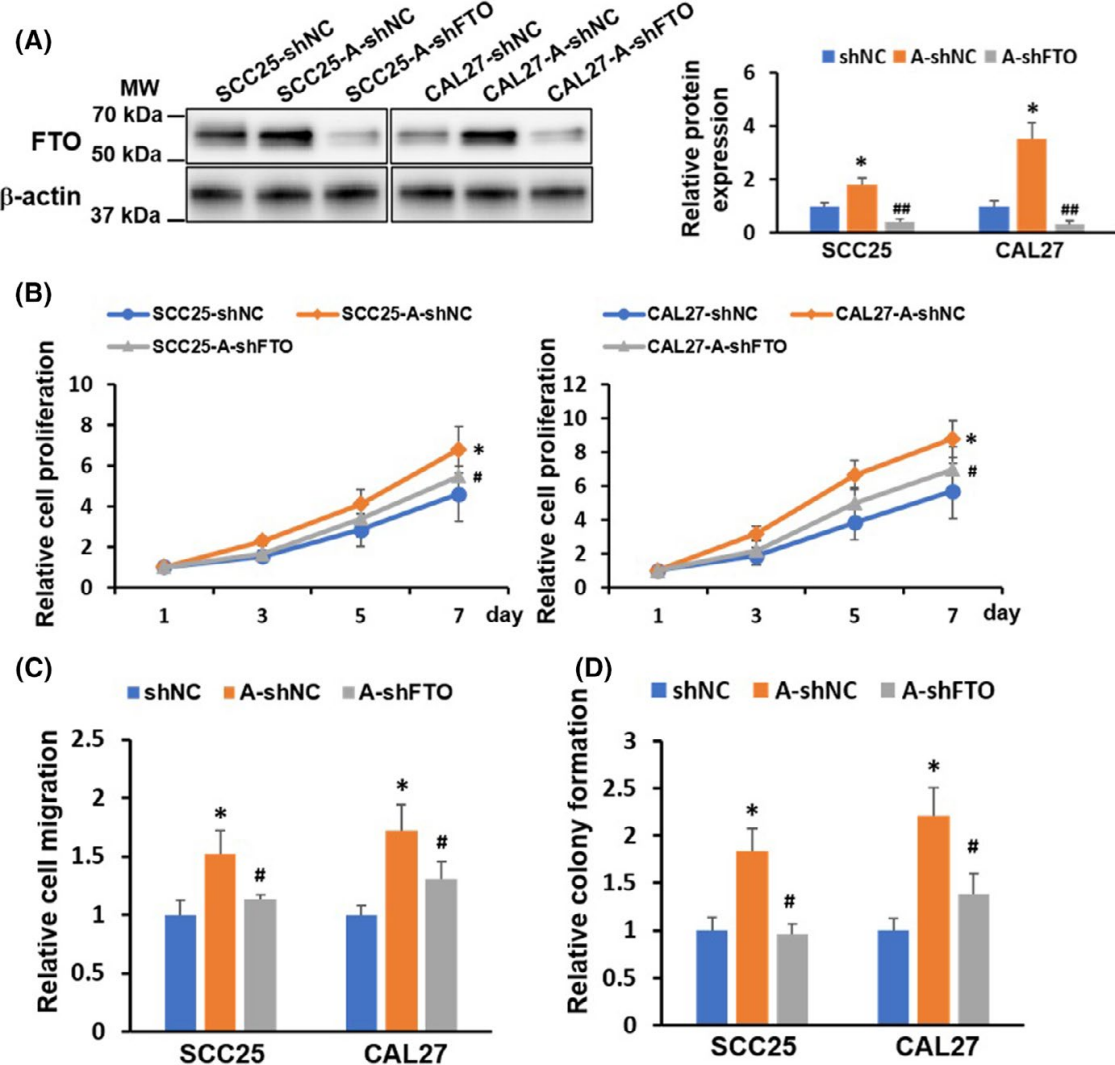
Knockdown of FTO reduced cell growth and migration of arecoline‐treated OSCC cells. Long‐term DMSO‐treated OSCC cell lines (refer to as SCC25, and CAL27), and arecoline (1μg/ml)‐treated OSCC cell lines (refer to as SCC25‐A, and CAL27‐A). SCC25, SCC25‐A, CAL27, and CAL27‐A cells transduced with lentivirus carrying shFTO or shNC (refer to as SCC25‐shNC, SCC25‐A‐shNC, SCC25‐shFTO, CAL27‐shNC, CAL27‐A‐shNC, and CAL27‐shFTO). (A) The protein levels of FTO in the indicated cells were measured by western blotting assay, and were quantified by Image J. (B) The cell proliferation of the indicated cells was assessed by CCK‐8 assay. (C) The cell migration of the indicated cells was tested by Boyden's chamber assay. (D) The colony formation of the indicated cells was measured by colony formation assay. Results are presented from three independent experiments. The molecular weight (MW) was depicted in the Figure. *p < 0.05 versus DMSO control group; #*p* < 0.05 shFTO versus shNC

To investigate the biological function of FTO in arecoline‐treated OSCC. SCC25‐A and CAL27‐A were infected with lentiviral vectors encoding shRNA targeting FTO (shFTO) or a nonsense control (shNC). The knockdown effect of FTO in SCC25‐A‐shFTO and CAL27‐A‐shFTO was confirmed by western blotting assay (Figure 3A). Results in Figure 3B–D demonstrated that arecoline‐treated OSCC (SCC25‐A‐shNC and CAL27‐A‐shNC) exhibited more vital ability in cell proliferation, cell migration, and colony formation than those in DMSO‐treated OSCC (SCC25‐shNC and CAL27‐shNC) (Figure 3B–D). The arecoline‐induced OSCC cell growth and migration effects were partially abrogated by knocking down FTO in SCC25‐A‐shFTO and CAL27‐A‐shFTO compared to SCC25‐shNC and CAL27‐shNC (Figure 3B–D).

### 3.4 FTO upregulation is critical for arecoline‐induced OSCC stemness and cisplatin resistance

**FIGURE 4:**
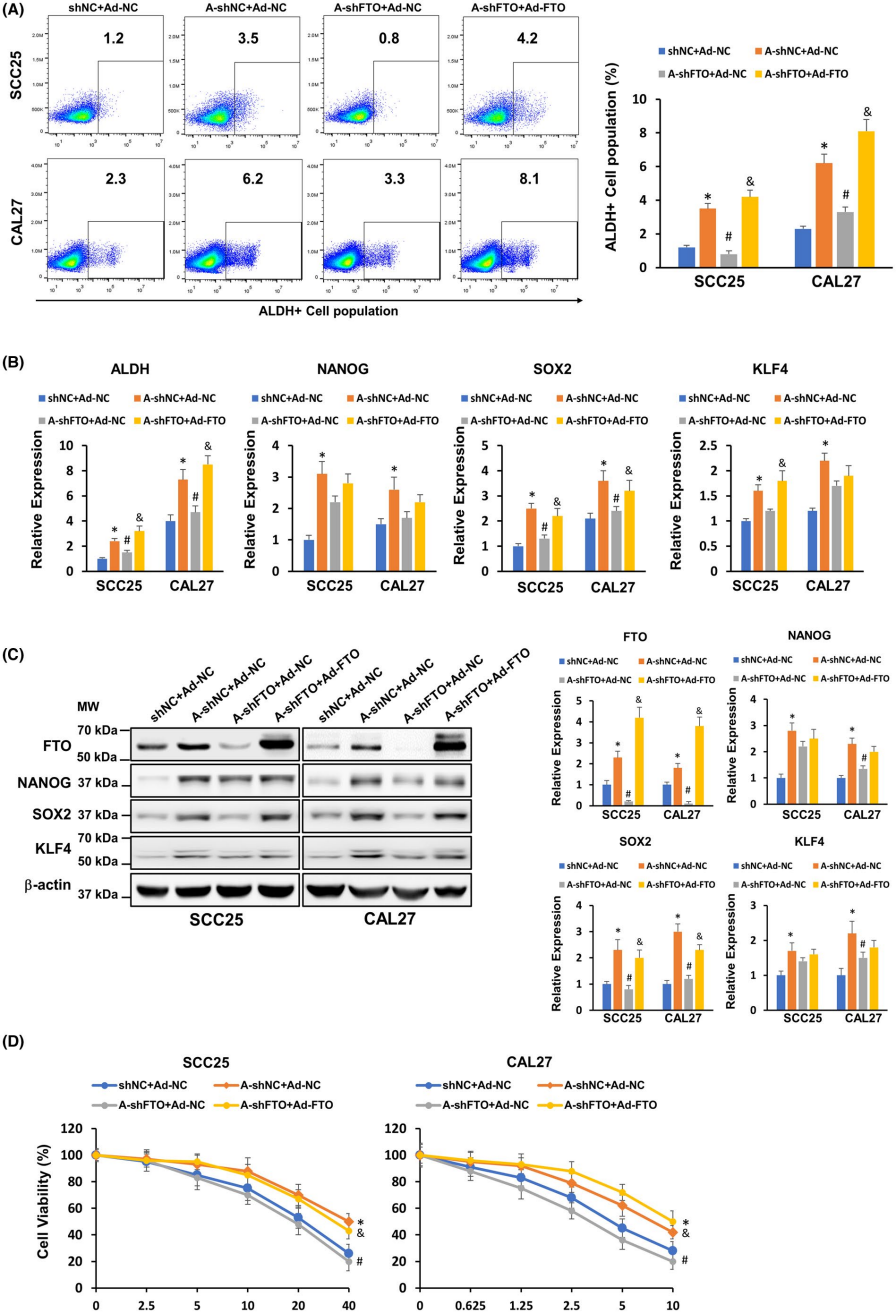
FTO plays a crucial role in arecoline‐induced stemness and cisplatin‐resistance of OSCC cells. (A) The ALDH‐positive population in SCC25‐shNC+Ad‐NC, SCC25‐A‐shNC+Ad‐NC, SCC25‐shFTO+Ad‐NC, SCC25‐shFTO+Ad‐FTO, CAL27‐shNC+Ad‐NC, CAL27‐A‐shNC+Ad‐NC, CAL27‐shFTO+Ad‐NC, and CAL27‐shFTO+Ad‐FTO cells were determined by flow cytometry analysis, and quantified by FlowJo software. (B) The expression levels of ALDH, NANOG, SOX2, and KLF4 in the indicated cells were detected by RT‐qPCR assay. (C) The protein levels of FTO, NANOG, SOX2, KLF4, and β‐actin in the indicated cells were measured by western blotting assay, and were quantified by Image J. (D) The indicated cells were treated with a serial dose of cisplatin for 24 h. The cell viability was measured by CCK‐8 assay. The molecular weight (MW) was depicted in the Figure. *p < 0.05 versus DMSO control group; # *p* < 0.05 shFTO versus shNC

A previous study showed that chronic arecoline treatment induces OSCC acquisition of cancer stemness.^15^ In line with this finding, we found that the ALDH‐positive population was upregulated in arecoline‐treated OSCC (SCC25‐A‐shNC and CAL27‐A‐shNC) and was downregulated in FTO knockdown OSCC (SCC25‐A‐shFTO and CAL27‐A‐shFTO) (Figure 4A). We further measured the expression of pluripotency factors, including NANOG, SOX2, and KLF4, in these OSCC cell lines. We found that both mRNA and protein levels of NANOG, SOX2, and KLF4 were upregulated in arecoline‐treated OSCC, and depletion of FTO significantly decreased at least one pluripotency factor in arecoline‐treated OSCC (Figure 4B,C). Moreover, we observed that arecoline‐treated OSCC was more resistant to cisplatin treatment than DMSO‐treated OSCC, and the cisplatin‐resistant ability of arecoline‐treated OSCC was impaired after FTO suppression (Figure 4D). To further confirm the role of FTO in the regulation of arecoline‐induced OSCC stemness and cisplatin resistance, the FTO rescue approach by adenoviral infection was applied. As shown in Figure 4C, the adenoviral particles carrying FTO cDNA infection resulted in substantial upregulation of FTO in SCC25‐A‐shFTO and CAL27‐A‐shFTO cells. Moreover, restoring FTO expression partially or completely rescued the FTO‐knockdown‐mediated inhibition effects on pluripotency factor expression and cisplatin‐resistant phenotype in SCC25‐A‐shFTO and CAL27‐A‐shFTO cells (Figure 4A–D). Taken together, these results suggested that FTO is positively associated with OSCC acquisition of cancer stemness and chemoresistance to cisplatin.

### 3.5 FTO is essential for arecoline‐promoted OSCC tumor growth

**FIGURE 5:**
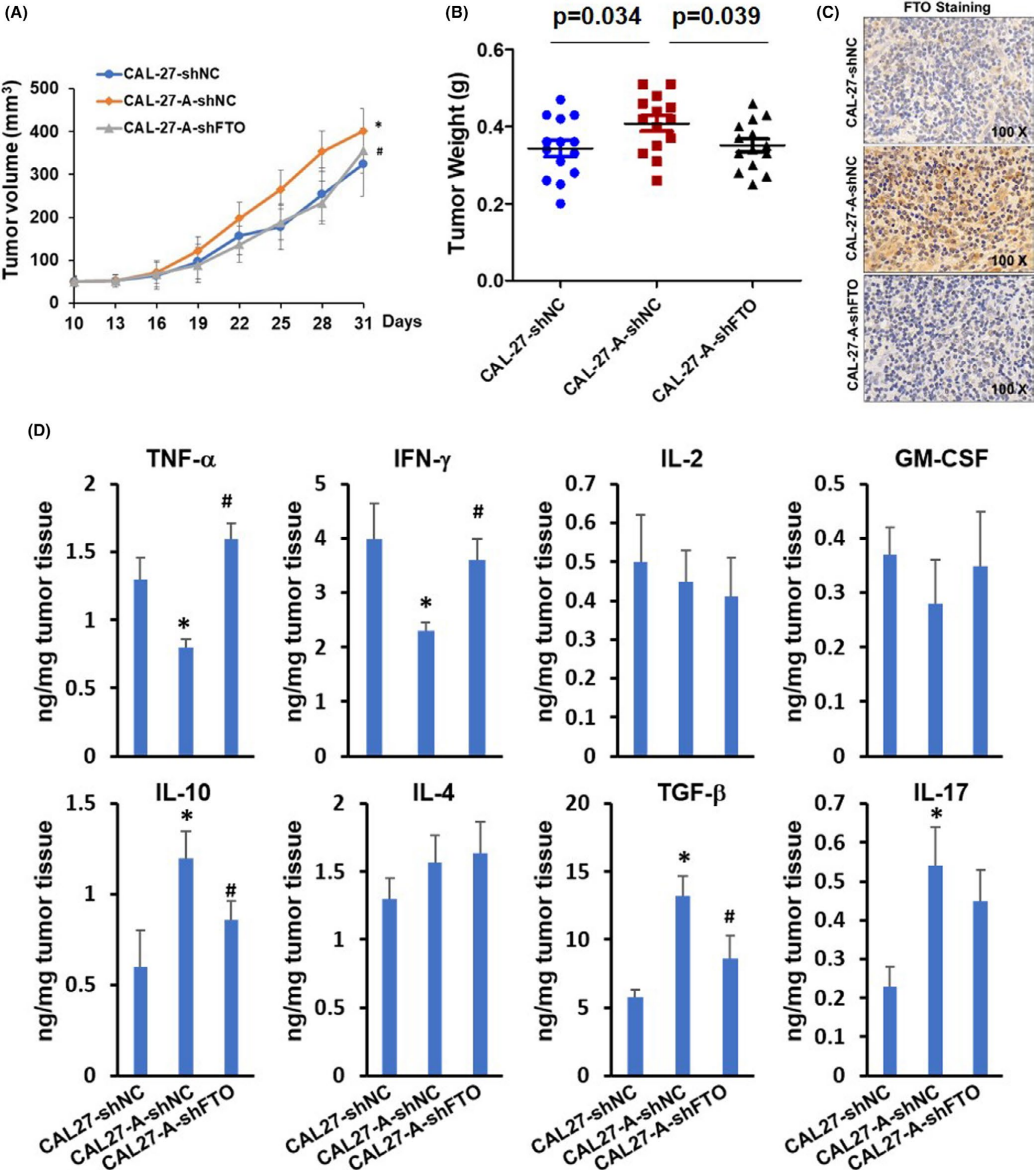
FTO silencing inhibited arecoline‐induced tumor growth and anti‐inflammatory cytokine expression in OSCC cells. (A,B) Mice were subcutaneously injected with CAL27‐shRNA, CAL27‐A‐shRNA, or CAL27‐A‐shFTO, and tumor volumes were measured every 3 days starting at 10‐day post tumor implantation (A). Tumor tissues were weight on 31‐day post tumor implantation (B). (C) The expression levels of FTO in tumor tissues from CAL27‐shRNA group, CAL27‐A‐shRNA group, and CAL27‐A‐shFTO group were determined by IHC staining. (D) The expression levels of TNF‐α, IFN‐γ, IL‐2, GM‐CSF, IL‐10, IL‐4, TGF‐β, and IL‐17 in tumor tissues were measured by ELISA assay. *p < 0.05 versus DMSO control group; #*p* < 0.05 shFTO versus shNC

BALB/c strain nude mice were subcutaneously injected with CAL27‐shRNA, CAL27‐A‐shRNA, or CAL27‐A‐shFTO, and tumor growth was monitored every 3 days starting at 10‐day post tumor implantation. As exhibited in Figure 5A,B, the CAL27‐A‐shRNA tumor grew faster and larger than CAL27‐shRNA tumor. The tumor growth between CAL27‐A‐shFTO and CAL27‐shRNA was comparable, suggesting that FTO silencing significantly reduced the tumor growth rate of CAL27‐A cells. Upregulation of FTO in CAL27‐A‐shRNA tumor compared to CAL27‐shRNA tumor and downregulation of FTO in CAL27‐A‐shFTO tumor compared to CAL27‐A‐shRNA tumor were confirmed by immunohistochemistry staining (Figure 5C). Immunosuppressive microenvironment is beneficial for tumor development. We determined the expression levels of pro‐inflammatory cytokines (TNF‐α, IFN‐γ, IL‐2, and GM‐CSF) and anti‐inflammatory cytokines (IL‐10, IL‐4, TGF‐β, and IL‐17) in tumors. As shown in Figure 5D, the TNF‐α and IFN‐γ levels were significantly decreased in CAL27‐A‐shRNA tumors compared to CAL27‐shRNA tumors. On the contrary, the IL‐10, TGF‐β, and IL‐17 were dramatically increased in CAL27‐A‐shRNA tumors. Interestingly, FTO depletion restored the expression levels of TNF‐α, IFN‐γ, IL‐10, and TGF‐β, but not on IL‐17 in CAL27‐A formed tumor. No significant differences were observed for the expression levels of IL‐2, GM‐CSF, and IL‐4 among three tumor groups.

### 3.6 FOXA2 negatively regulates FTO expression in OSCC

**FIGURE 6:**
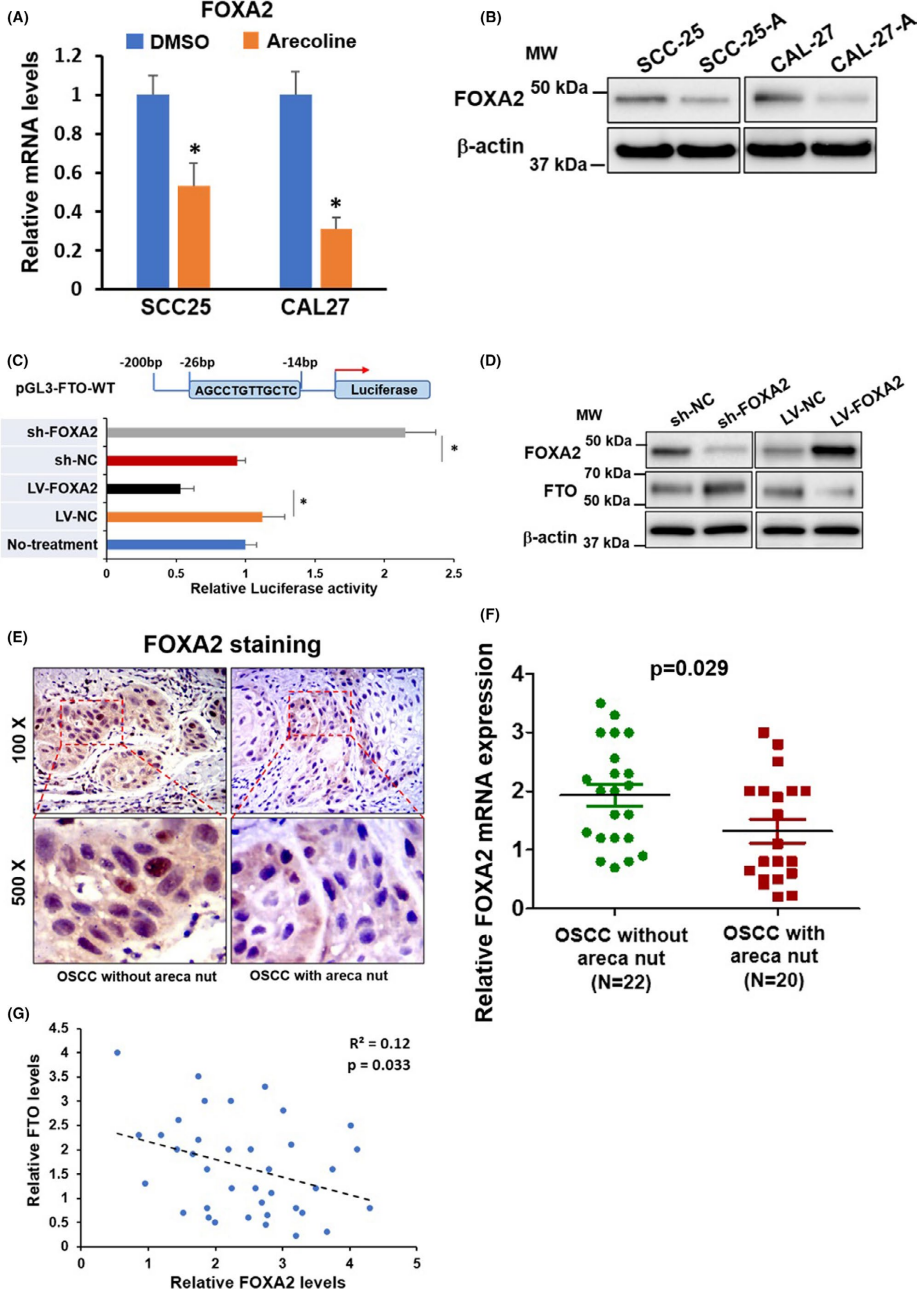
FTO is negatively regulated by FOXA2 in OSCC. (A,B) The mRNA and protein levels of FOXA2 were detected by RT‐qPCR assay (A), and western blotting assay (B), respectively. (C,D) 293T cells were transduced with lentiviral particles carrying FOXA2 gene or shFOXA2 (Short hairpin RNA targeting FOXA2, and control lentivirus (LV‐NC, or sh‐NC) and then co‐transfected with pGL3‐FTO and pRT‐TK vectors (Promerga). The luciferase activities were determined 24h after transfection (C). The protein levels of FOXA2, FTO, and β‐actin were measured by western blotting assay (D). (E,F) The expression levels of FOXA2 in OSCC without areca nut (*n* = 22), and OSCC with areca nut (*n* = 20) were determined by IHC staining (E), and RT‐qPCR assay (F). (G) The correlation of FOXA2 and FTO was analyzed by Spearman correlation analysis. The molecular weight (MW) was depicted in the Figure. *p < 0.05 versus control group

Transcription factor FOXA2 is reported to regulate FTO expression negatively.^16^ We discovered that the mRNA and protein levels of FOXA2 were significantly downregulated in SCC25‐A and CAL27‐A compared to SCC25 and CAL27. The opposite change pattern between FOXA2 and FTO indicated that the downregulation of FOXA2 might be responsible for FTO upregulation in SCC25‐A and CAL27‐A (Figure 6A,B). To address this question, we applied a luciferase reporter assay. As represented in Figure 6C, overexpression of FOXA2 suppressed, whereas suppression of FOXA2 enhanced the luciferase activities of the Luc‐vector containing a FOXA2 binding site in its promoter region. Indeed, we exhibited that FOXA2 knockdown led to upregulation of FTO protein expression and vice versa (Figure 6C,D). The IHC results in Figure 6E and RT‐qPCR results in Figure 6F manifested that FOXA2 expression levels were substantially downregulated in OSCC samples with chronic areca nut exposure compared to OSCC samples without areca nut exposure (Figure 6E,F). Notably, the Figure 6G elucidated that the expression of FTO and FOXA2 was inversely correlated in 42 OSCC samples. Collectively, these data suggested that FTO was negatively regulated by FOXA2 both in vitro and in vivo.

## 4 DISCUSSION

Accumulating studies have demonstrated that arecoline, a primary active alkaloid in areca nut, is a potent carcinogen for the development of oral submucous fibrosis and oral cancer.^17^ Numerous studies have comprehensively investigated the acute cytotoxic and genotoxic effects of arecoline on oral fibroblast, keratinocytes, and cancer cells.^7^ However, the information concerning the long‐term effects of arecoline on oral cells is lacking. We exhibited that low concentration (e.g., 1 µg/ml, and 2.5 µg/ml) of arecoline long‐term treatment, which may reflect the daily areca nut chewing habit, significantly promoted cell proliferation, migration, stemness, and cisplatin‐resistance of OSCC cells, suggesting chronic arecoline exposure transformed OSCC cell lines to more tumor aggressiveness. Our primary data is consistent with others’ findings. Multiple studies have shown that high doses of arecoline cause toxic effects on various cell types and organs, even death of animals.^10^, ^18^, ^19^ In contrast, low doses of arecoline increased cell proliferation of oral fibroblasts and OSCC cell lines. A recent paper revealed that low dose, long‐term arecoline treatment enhanced stemness, chemoresistance, and oncogenicity of OSCC cells.^20^

In recent years, increasing literature manifested the critical roles of m6A mechanism in human cancer, including OSCC. Three major m6A regulators have been well‐characterized in the past years, which include methyltransferase (writers), demethylase (erasers), and methylation recognition (reader) enzymes.^12^, ^13^ FTO, a member of demethylase, has been recognized as a key enzyme in the regulation of tumor occurrence, development, and progression.^21^ Overexpression of FTO has been observed in multiple human cancer and is generally recognized as an oncogene in various types of cancers, including leukemia, brain, breast, gastric, lung, and cervical cancer.^22^, ^23^, ^24^, ^25^ Li et al. first reported upregulation of FTO in a subtype of acute myeloid leukemia cells and forced expression of FTO enhanced leukemogenesis in mice.^26^ Cui et al. showed that targeting FTO using FTO inhibitor exerted strong tumor suppression effects on mice with glioblastoma.^27^ Upregulation of FTO has also been reported to facilitate the development of lung adenocarcinoma and hepatocellular carcinoma.^28^, ^29^ The role of FTO in oral cancer is not fully understood. We revealed that FTO was notably upregulated in OSCC, especially in OSCC patients with areca nut chewing habit. Our data suggested that FTO not only plays an oncogenic role in oral cancer development, but also plays a pivotal role in arecoline‐induced tumorigenesis of oral cancer. Indeed, we demonstrated that depletion of FTO significantly reduced arecoline‐transformed oral cancer cell proliferation, migration, stemness, and cisplatin‐resistance in vitro, as well as tumor growth in vivo. Moreover, restoration of FTO expression was able to partially or completely rescue FTO‐knockdown‐induced inhibition effects on pluripotency factor expression and cisplatin‐resistant phenotype in arecoline‐treated oral cancer cells, confirming the crucial oncogenic roles of FTO in arecoline‐treated oral cancer cells.

Since the first discovery of FTO in mouse in 1999, the functional roles of FTO have been extensively investigated in obesity, Alzheimer's disease, and recently in cancers.^30^, ^31^ However, the transcriptional mechanism regulating the expression of FTO is relatively lacking. Guo et al. identified that a transcription factor‐FOXA2 negatively regulates FTO expression in 293 cells.^16^ We found that the expression levels of FOXA2 were downregulated in long‐term arecoline‐treated oral cancer cells. Forcing FOXA2 expression inhibited, whereas silencing FOXA2 expression promoted FTO expression in oral cancer cells, proving that FTO is negatively regulated by FOXA2. Importantly, we illustrated that the expression of FOXA2 was downregulated in OSCC tissues from patients with areca nut chewing habit compared to OSCC tissues from patients without areca nut exposure, and the expression levels of FOXA2 and FTO were negatively correlated in OSCC tissues, confirming that FOXA2 negatively regulates FTO expression in vivo.

Although our results revealing the oncogenic roles of FTO in arecoline‐induced OSCC tumorigenesis, many questions are not addressed and urgently need further investigation in the future. For example, which signaling pathway is mediated by FTO in arecoline‐treated OSCC cell lines? Whether other mechanisms (e.g., transcription factor, microRNAs, and epigenetics) are involved in regulating FTO expression? How FTO affects mRNA modification to regulate key gene function in arecoline‐treated OSCC cell lines? More importantly, to further investigate the role of FTO in the regulation of the interaction between tumors and immune cells in tumor microenvironment, a mouse model with full immunocompetence (e.g., C57B/6 mouse strain) is needed in future studies. The answers to these questions may contribute to the development of novel methods to restore the dysregulation of FTO in human cancers.

Our current study demonstrated that FTO plays a vital role in chronic low‐dose arecoline exposure‐promoted stemness, chemoresistance, and oncogenicity of OSCC cells. Targeting FTO may serve as a new strategy to treat OSCC patients, especially those with areca nut chewing habits.

## DISCLOSURE STATEMENT

The authors declare no conflict of interest.

## Supporting information

Figure S1.

Table S1.

## Data Availability

All data generated or analyzed during this study are included in this article and its supplementary information files.
