# Correction to Fat mass and obesity‐associated protein regulates tumorigenesis of arecoline‐promoted human oral carcinoma

**DOI:** 10.1002/cam4.7433

**Published:** 2024-06-24

Li X, Xie X, Gu Y, Zhang J, Song J, Cheng X, Gao Y, Ai Y. Fat mass and obesity‐associated protein regulates tumorigenesis of arecoline‐promoted human oral carcinoma. Cancer Med. 2021 Sep;10(18):6402–6415. https://doi.org/10.1002/cam4.4188

Concerns were raised by a third party regarding the * and # markers included in Figures 3B and 5A, as both indicate *p* < 0.05. Although there is an explanation in the figure legend that *p < 0.05 versus DMSO control group and #*p* < 0.05 shFTO versus shNC, the authors accept the suggestion that the labels could be improved to avoid confusion. The corrected figures are provided below.

The authors apologize for any confusion caused.

FIGURE 3B:

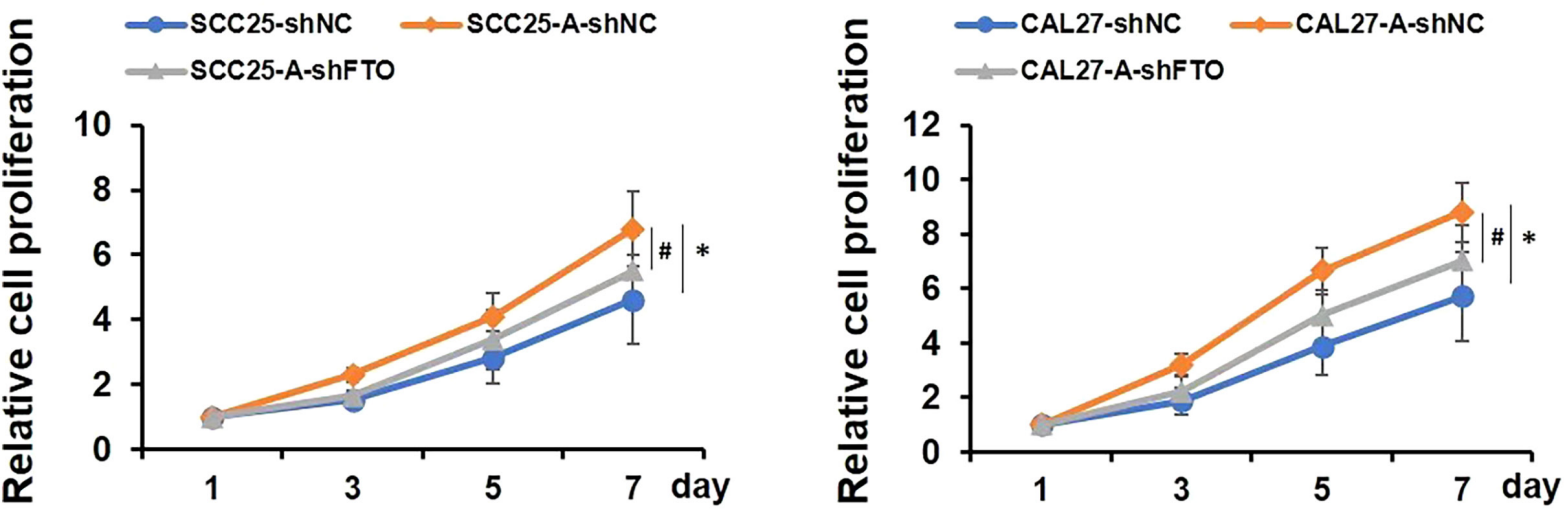

Updated figure legend: *p < 0.05 indicates A‐shNC group versus shNC control group, and #*p* < 0.05 indicates A‐shFTO group versus A‐shNC group.

FIGURE 5A:

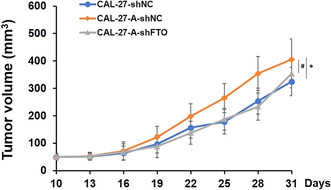

Updated figure legend: *p < 0.05 indicates A‐shNC group versus shNC control group, and #*p* < 0.05 indicates A‐shFTO group versus A‐shNC group.

