# Supplementary figures and images for: Fat mass and obesity‐associated protein regulates tumorigenesis of arecoline‐promoted human oral carcinoma
Source: Cancer Med. 2021 Aug 11;10(18):6402–15. doi: 10.1002/cam4.4188 (PMC8446412; doi:10.1002/cam4.4188)

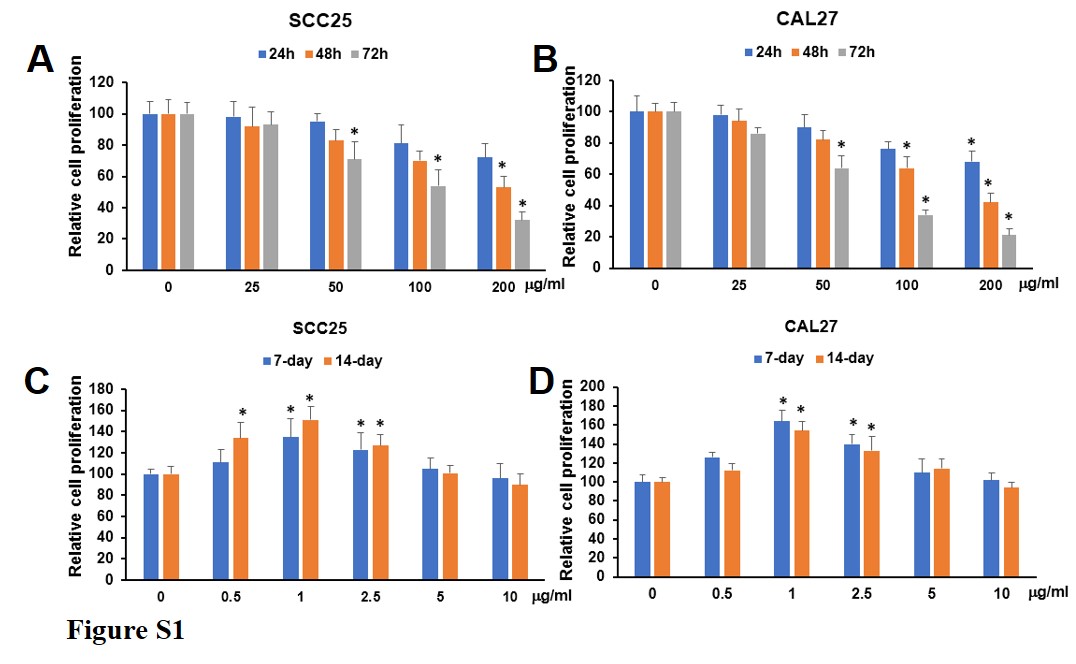

Supplement: Supplementary file 1 — Figure S1. [file CAM4-10-6402-s002.jpg]

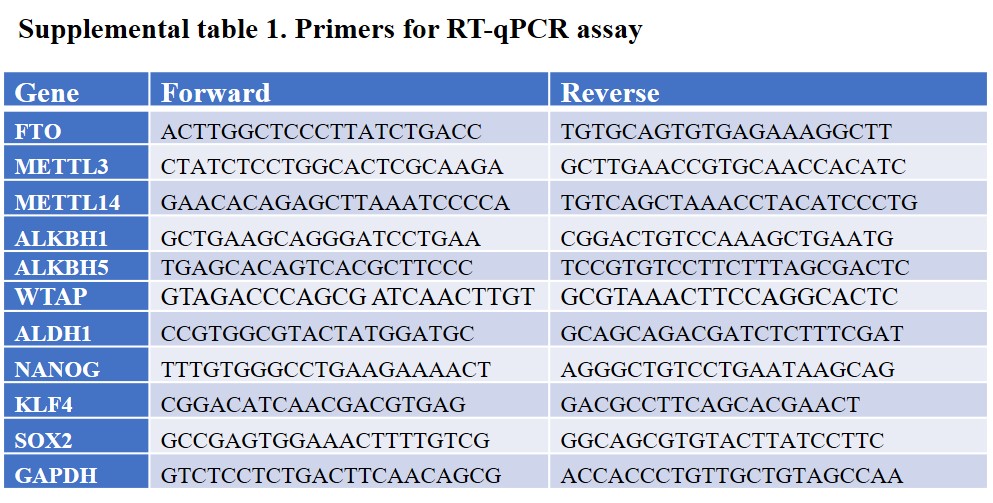

Supplement: Supplementary file 2 — Table S1. [file CAM4-10-6402-s001.jpg]
